# Performance evaluation and quality assurance of Varian enhanced dynamic wedges

**DOI:** 10.1120/jacmp.v7i1.2170

**Published:** 2006-02-21

**Authors:** Parham Alaei, Patrick D. Higgins

**Affiliations:** ^1^ Department of Therapeutic Radiology‐Radiation Oncology University of Minnesota Minneapolis Minnesota 55455 U.S.A.

**Keywords:** dynamic wedge, enhanced dynamic wedge, quality assurance

## Abstract

Dynamic wedges have been used in clinical practice for many years. Obvious superiority of dynamic over physical wedges is accompanied by the increased overhead involved in verifying the accuracy and reliability of their use. Contrary to very limited QA required to ensure proper functioning of the physical wedges, dynamic wedges, like any other dynamic treatment, require a robust QA program. This work expands upon previous suggestions and describes a comprehensive QA program for Varian enhanced dynamic wedges (EDWs) and presents the results of an 18‐month evaluation of these wedges. The QA program includes daily, monthly, and yearly tests and individual treatment QA at the onset of use of the EDWs. The results of the 18‐month evaluation show reproducibility in the wedge factors of better than 1% and in dose profiles of better than 2% on a monthly basis. Daily output measurements are generally within 2% of expected values.

PACS numbers: 87.53.Xd, 87.56.Fc

## I. INTRODUCTION

The enhanced dynamic wedge (EDW) is an option on newer Varian LINACs. In this mode of treatment, one of the upper jaws sweeps across the field from its maximum open position to within 0.5 cm of the opposite jaw, creating a wedged beam profile. In order to deliver a dynamically wedged field, the length of the treatment field is divided into 20 segments, and the speed of the moving jaw and the dose rate within each segment are controlled based on a calculated segmented treatment table (STT) generated by the LINAC computer. The STT is essentially a table of positions of the moving jaw versus the cumulative monitor units delivered at each position. The STT for a particular wedged delivery is a product of weighted averaging between an open‐field STT and a 60° “golden” STT. The details of STT generation and delivery have been explained by Varian.^(1)^ This study is based on data collected on a Varian 21EX LINAC with dynamic wedge angles of 10°, 15°, 20°, 25°, 30°, 45°, and 60° and photon energies of 6 MV and 10 MV.

Like any other mode of dynamic treatment, use of EDWs should be accompanied by a rigorous QA program. The QA procedures described here have been implemented to assure proper functioning of the EDWs and can be easily performed in a clinical environment. This work is meant to be an update to previous reports on EDW implementation and QA, such as those by Moeller et al.^(2)^ and Koken et al.^(3)^ Moeller et al.^(2)^ have proposed a number of daily and monthly checks for EDWs. This work expands upon those recommendations and adds other tasks such as STT analysis and more extensive monthly and annual wedge factor measurements. We have also chosen to use the 30° (instead of the 60°) wedge angle for daily QA. Finally, we present the results of an 18‐month evaluation of the EDWs using the QA procedures described here.

## II. METHODS

The proposed QA of EDWs includes daily, monthly, and yearly tasks, as well as individual treatment verifications. The daily QA includes delivery of one EDW field per beam energy as part of the morning QA of the LINAC and recording the value of the central axis reading at a standard depth. The monthly QA includes measuring a sample of wedge factors, obtaining a sample of beam cross profiles, and saving a sample of dynalog files generated by the LINAC. Dynalog files contain information such as date and time of treatment, energy, monitor units, orientation, and type of treatment, as well as the STT calculated for the treatment and the actual delivered STT. The annual QA includes a larger sampling of wedge factors, similar to that for physical or hard wedges, and an analysis of saved dynalog files. The individual treatment verification or “per patient” QA includes visual inspection of the wedge direction at the conclusion of treatment and may only be necessary at the onset of the use of dynamic wedges.

### A. Daily QA

Two EDW fields, one for each photon energy, have been added to the morning photon QA list. The wedge angle of 30° was chosen for this because delivering a 30° wedged field acts as a check of the integrity of the algorithm, since a “weighting” of the open and golden STTs must be performed to deliver this field. The central axis wedge factor can be obtained by dividing the “wedged” reading by the “open” reading. This value is used as a constancy check and is proportional to the clinically used wedge factors measured under standard conditions. Similar to open field constancy measurements, a limit of ±3% must be satisfied pursuant to TG‐40 specifications^(4)^; otherwise, the treatments will not commence pending a review by physicists.

The morning output check device used here is the VeriDose system (Cardinal Health, Cleveland, OH) and is calibrated on a bimonthly basis. This device is diode‐based, thus eliminating the need for temperature and pressure corrections. The diodes are beneath 2.3 cm water equivalent buildup; no additional buildup was used for measurements. A field size of $20\times 20\;{\text{cm}}^{2}$ is used for the morning output checks.

### B. Monthly QA

Monthly QA consists of wedge factor and profile measurements. In addition, a number of dynalog files are saved in order to create a dataset for future analysis. Wedge factors are measured for a rotating selection of fields, of varying field sizes and energies, month to month. All the wedge factors are measured in a water phantom under standard measurement conditions (100 cm source‐to‐surface distance (SSD), 10 cm depth). A total of 14 wedge factors (one per wedge angle per energy) are measured each month. The wedge factors are then compared to those in the dosimetry tables, as obtained at the time of commissioning. Linear interpolation is used to obtain wedge factors for field sizes not measured at the time of commissioning.

Profile measurements are performed using the Profiler (Sun Nuclear, Melbourne, FL) for a fixed field size and all the wedge angles and energies. The purpose of this test is to check the constancy of the delivery of EDW fields. The Profiler consists of a linear diode array of 46 detectors with 0.5 cm spacing and with a 1‐cm water equivalent internal buildup. Additional buildup plates can be added to bring the scan plane to the desired depth. Our experiment with the addition of buildup plates showed no improvement in the reproducibility of the profiles for these energies (6 MV and 10 MV); thus, we chose not to add plates for ease of measurement. The Profiler's performance has been evaluated by Zhu et al.^(5)^ The maximum usable field size at 100 cm SSD is $20\times 20\;{\text{cm}}^{2}$. All the Profiler measurements performed here were at 100 cm SSD with a $20\times 20\;{\text{cm}}^{2}$ field size without any additional buildup added to the device.

During the delivery of a dynamically wedged field, the LINAC constantly checks the dose delivered against the desired delivery; that is, the delivered (or actual) STT is compared to the calculated STT, and an interlock is triggered should a discrepancy greater than 0.5 cm spatially or 0.3 monitor units dosimetrically be observed.^(1)^ These data are saved in the dynalog file. The LINAC stores up to 199 dynalog files, constantly overwriting the older ones. Thus, in order to analyze them, they need to be frequently backed up on a secondary storage device (i.e., a floppy disk). Due to the sheer volume of dynamic treatments delivered, it is impractical, and unnecessary, to save all the dynalogs. We, however, maintain a consistent set of saved dynalogs, by delivering a set of dynamic wedge treatments in the clinical mode using the same field size, energy, wedge angle, and monitor units, on a monthly basis, and saving the dynalog files afterward. This set consists of only two profiles, a $30\times 20\;{\text{cm}}^{2}$ field (largest deliverable field size in the *Y*‐direction), 30° EDW, for both energies. The saved dynalogs are used for the annual review and may be used for diagnosing problems that may manifest over time.

### C. Annual QA

Annual QA is similar to that used for physical wedges, with wedge factors measured for all wedges and both energies for a $10\times 10\;{\text{cm}}^{2}$ field and compared with commissioning data. Again, all measurements are performed in a water phantom at 100 cm SSD and 10 cm depth. The analysis of the dynalog files saved on a monthly basis is done during the annual QA by plotting the delivered STTs and comparing them.

### D. Individual Patient QA

Patient QA is an essential part of QA at the commencement of EDW implementation, and unfamiliarity of the therapists with the EDW may add to its importance. Normally, the matching of wedge direction between the plan, chart, and record‐and‐verify system is checked by the dosimetrist and physicist. It is, however, good practice to ask the therapists to confirm the orientation due to its importance in proper delivery of wedged fields. In the case of physical or hard wedges, the heel and toe direction of the wedge is usually checked by the therapists before the commencement of the first treatment. For EDWs, the verification of wedge direction prior to treatment can only be done by checking the wedge direction graphics on the collimator head. Posttreatment, the wedge direction can be verified by visually inspecting the location of the “light strip.” The light strip, which indicates the final position of the moving jaw, is the “toe” direction of the wedge that can be checked against the documentation in the chart. The therapists need to be trained to enter the room following the conclusion of treatment of each field to check the light strip on the patient's skin. This is particularly important when beginning to use EDWs, as a secondary check to proper transfer of information from the treatment‐planning system to the record‐and‐verify system, and can be eliminated once it is clear that no problems exist.

All the QA tasks performed are summarized in Table 1.

**Table 1:** Summary of QA tasks performed.

Frequency	Task
Daily	EDW functionality check
Monthly	Wedge factor measurements Profile measurements Dynalog (STT) saving
Annual	Wedge factor measurements Dynalog (STT) analysis
Patient	Wedge direction verification (intial use only)

## III. RESULTS AND DISCUSSION

**Figure 1:**
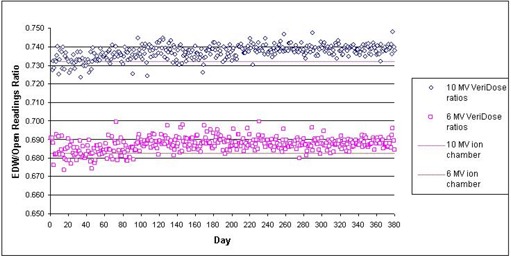
Variation of daily dynamic wedge factors over an 18‐month period, 6 MV and 10 MV, $20\times 20\;{\text{cm}}^{2}$ field size, 30° EDW as compared to ion chamber‐measured wedge factors.

**Figure 2:**
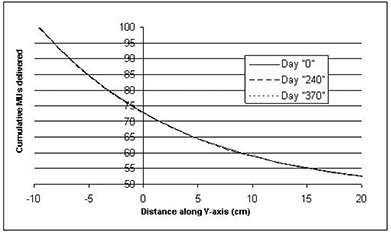
Comparison of delivered segmented treatment tables (STTs) at days 0, 240, and 370 (corresponding to Fig. 1), 10 MV, $20\times 30\;{\text{cm}}^{2}$ field size, 30° EDW.

Figure 1 shows the results of daily constancy checks over an 18‐month period. The values are the EDW‐to‐open readings ratios for each energy, thus eliminating the day‐to‐day variations in the QA phantom's diode response or slight fluctuations in machine output. As seen here, the daily constancy check shows a stable delivery of the wedged field with a reproducibility of better than 1%. The ion chamber‐measured wedge factors at the same depth (2.3 cm) are also plotted for comparison as straight lines. As seen in the figure, the variation of these values over time is within 2% of the ion chamber‐measured factor, although there appears to be a slight upward trend over time. Over the same period of time (days 0, 240, and 370), we have plotted the delivered STTs from the dynalog files in Fig. 2. As seen here, the three curves are virtually superimposed, verifying the consistency of dose delivery over the evaluation period.

Monthly wedge factor measurements over the 18‐month period show an average percentage difference of 0.31(±0.29) for the 6‐MV and 0.71(±0.35) for the 10‐MV beam as compared to the values obtained at commissioning.

**Figure 3:**
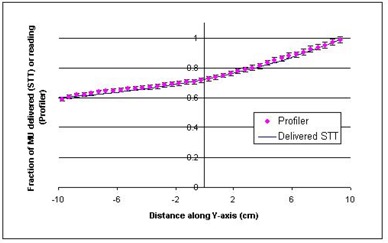
Comparison of a delivered segmented treatment table (STT) and Profiler plot of the same dynamic wedge delivery, 6 MV, $20\times 20\;{\text{cm}}^{2}$, 25° EDW. The error bars represent a ±2% variation in Profiler values and are drawn to illustrate the degree of agreement between the two datasets.

**Figure 4:**
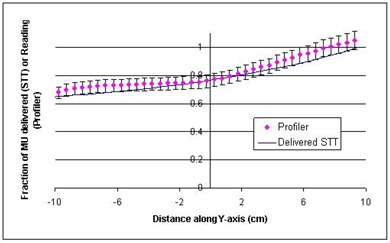
Comparison of a delivered segmented treatment table (STT) and Profiler plot of the same dynamic wedge delivery, 10 MV, $20\times 20\;{\text{cm}}^{2}$, 25° EDW. The error bars represent a ±6% variation in Profiler values and are drawn to illustrate the degree of agreement between the two datasets.

Figures 3 and 4 are comparisons of Profiler plots and the delivered (actual) STTs, extracted from the dynalogs. The data presented in these figures are matched at the central axis. The quantities compared here are related but not the same. The STT is a measure of monitor units delivered per segment, and the Profiler plot is a measure of dose measured at the detector plane of the Profiler, at the depth of $d_{\max}$. So some difference between the two quantities is expected, mostly due to scattering off the collimator jaws and variations in partial transmission through the moving jaw at various positions; otherwise, they should follow the same trend. As seen in the figures, the maximum difference between STT and dose profiles is about 2% for the 6‐MV beam and 6% for the 10‐MV beam. The error bars in these figures do not represent measurement uncertainty; rather, they are drawn to illustrate the degree of agreement between the sets of data. We have also found that larger field sizes exhibit a greater difference between these two curves. In general, however, the monitor units delivered as a function of jaw position (delivered STT) correlate consistently and reproducibly with the dose profile measured.

**Figure 5:**
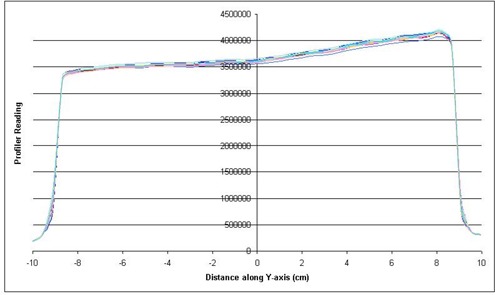
Intercomparison of wedge profiles over an 18‐month period, 6 MV, $20\times 20\;{\text{cm}}^{2}$, 10° EDW.

**Figure 6:**
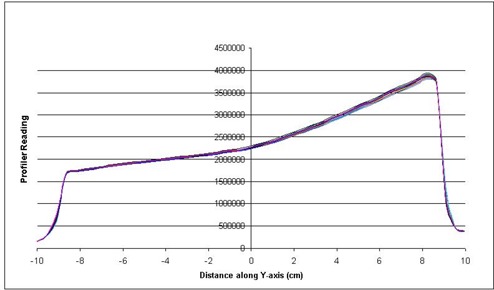
Intercomparison of wedge profiles over an 18‐month period, 10 MV, $20\times 20\;{\text{cm}}^{2}$, 45° EDW.

The reproducibility of the dose profiles is shown in Fig. 5 for the 6‐MV beam, 10° EDW and Fig. 6 for the 10‐MV beam, 45° EDW. These figures are an intercomparison of the profiles over the 18‐month period. As seen in the figures, the profiles are almost identical over time with a variation of less than 2%.

## IV. CONCLUSION

A comprehensive QA procedure for enhanced dynamic wedges has been proposed. Most of these QA routines can also be applied to the dynamic wedges available on other LINACs. An evaluation of the performance of these wedges is also presented. We have found very good reproducibility of these dynamic treatments over an 18‐month period for the 21ex accelerator, as well as accuracy and repeatability of the STT generation and delivery. We have outlined a workable QA procedure for this dynamic modality. The daily and monthly tests discussed here (daily wedge factor check, monthly wedge factor and profile measurements) should be adequate to detect any potential problems in the delivering of an EDW field.
